# Does Gamma-Glutamyl Transpeptidase Serum Level Play a Role as a Prognostic Marker in Biliary Atresia, an Immune-Mediated Cholangiopathy in Children?

**DOI:** 10.3390/jcm15010062

**Published:** 2025-12-22

**Authors:** Alina Grama, Alexandra Mititelu, Gabriel Benţa, Tudor Lucian Pop

**Affiliations:** 1 Second Pediatric Discipline, Department of Mother and Child, Iuliu Haţieganu University of Medicine and Pharmacy, 400177 Cluj-Napoca, Romania; gramaalina16@elearn.umfcluj.ro (A.G.); tudor.pop@umfcluj.ro (T.L.P.); 2 Second Pediatric Clinic, Center of Expertise in Pediatric Rare Liver Diseases, Emergency Clinical Hospital for Children, 400177 Cluj-Napoca, Romania

**Keywords:** gamma-glutamyl transpeptidase, biliary atresia, neonatal cholestasis, children, prognostic

## Abstract

**Background**: Biliary atresia (BA) is a rare, immune-mediated cholangiopathy in children and a leading cause of neonatal cholestasis and pediatric liver transplantation (LT). Gamma-glutamyl transpeptidase (GGT) is commonly elevated in BA and is used as a diagnostic marker; however, recent studies have suggested that a subset of BA patients present with normal or low GGT levels, potentially indicating a more severe disease course. **Methods**: This retrospective study evaluated the prognostic value of serum GGT levels in 47 children diagnosed with BA at a single center over 15 years. Patients were stratified by GGT levels at diagnosis, and outcomes were compared, including survival with native liver, need for LT, and mortality. GGT thresholds of 200 U/L and 300 U/L were used to define normal and elevated levels. **Results**: The study found that 12% of patients had normal or low GGT at diagnosis. Still, there were no statistically significant differences in age at diagnosis, severity of liver fibrosis, age at Kasai portoenterostomy (KPE), or overall outcomes between low and high GGT groups. Although patients with lower GGT tended to require LT at a younger age, this difference was not significant. Receiver operating characteristic (ROC) curve analysis showed that GGT levels at diagnosis, before, and after KPE were not reliable predictors of outcome. **Conclusions**: The findings contrast with some previous reports suggesting that low GGT is associated with a worse prognosis. Low GGT level alone should not delay diagnosis or surgical intervention in suspected BA. Early referral for KPE remains critical, and patients with low GGT may benefit from earlier LT evaluation. Larger, multicenter studies are needed to clarify the prognostic role of GGT in BA.

## 1. Introduction

Gamma-glutamyl transpeptidase (GGT) is a heterodimeric protein, located on the surface of the epithelial cells of tissues involved in the processes of secretion or absorption (bile ducts, kidneys, bowel, pancreas, and seminal vesicles) [1]. The highest concentration of GGT is found in hepatocytes and bile duct epithelial cells, and it is a sensitive indicator of liver disease, particularly hepatobiliary obstruction [2].

GGT is an essential blood test in evaluating neonatal cholestasis (NC). In most cases of intra- or extrahepatic cholestasis, GGT levels are elevated. Some rare cholestatic disorders, such as progressive familial intrahepatic cholestasis (PFIC) types 1 and 2, bile acid synthesis disorders (BASDs), and tight-junction protein type 2 deficiency, evolve with normal or low GGT [3]. Biliary atresia (BA) is a rare condition that falls under the category of immune-mediated cholangiopathies, along with neonatal sclerosing cholangitis (NSC) and primary sclerosing cholangitis (PSC) [4]. The disease is an important cause of NC and currently represents the main indication for liver transplantation (LT) in children [4,5]. In BA, the GGT level is often pathologically increased due to extrahepatic bile duct inflammation and obstruction, or secondary to compensatory intrahepatic bile duct proliferation, a histopathological sign of this condition [6]. Recent studies have reported a paradoxical subset of patients with normal or low GGT levels, mimicking genetic progressive cholestasis and delaying diagnosis. There are reports of a correlation between normal GGT levels and severe forms of BA, with an unfavorable disease course [6,7,8,9].

Our study aimed to determine whether there is an association between normal GGT levels and the severity of BA, as other recent papers claimed, and to verify whether GGT thresholds predict transplant-free survival in patients with or without Kasai hepatportoenterostomy (KPE). Additionally, we aimed to eventually propose a GGT-based algorithm to personalize monitoring and therapeutic strategies, thereby improving outcomes in these patients.

## 2. Materials and Methods

We conducted a retrospective study involving 47 children diagnosed with BA and followed our unit over 15 years, from January 2015 to December 2024. All of them were diagnosed with BA based on clinical manifestations (jaundice, acholic stools), associated with abdominal ultrasound and with abnormal intraoperative cholangiogram and histological proven obstruction of extrahepatic bile ducts (cellular necrosis, bile duct proliferation, ductular reaction defined as increased ductular profiles at the limiting plate or interface, and portal fibrosis) [10]. We excluded from this study all cases with other causes of NC and those with insufficient medical records.

The patients’ medical histories were obtained from their hospital records in the archives. We analyzed the demographic characteristics of patients (gender, age at diagnosis, age at KPE intervention if performed), nutritional status (per Centers for Disease Control and Prevention criteria), clinical parameters, comorbidities, and familial history of cholestatic liver disease. We studied GGT values at diagnosis, before and after KPE, and at 3 and 6 months post-KPE. Also, children without KPE were studied at the time of diagnosis; at ages of 3, 6, and 9 months; or before and after LT. To evaluate the outcomes, we classified patients into two groups: those with good evolution, who survived with the native liver, and those with unfavorable evolution, who required LT or had a fatal outcome. Children with KPE were subclassed in two other groups: those with complete restoration of bile flow (complete clearance of jaundice, resolution of acholic stools and reduced level of direct bilirubin to DB < 2mg/dL or smaller than 20% of total bilirubin (TB)) and those in which jaundice persisted after KPE and DB remained elevated at >2 mg/dL or greater than 20% of total bilirubin [3]. Normal GGT was defined as <200 U/L based on our laboratory’s reference range. We analyzed the data by GGT level, categorizing it as normal or high. To compare our data with other studies in the literature, we also classified other groups as follows: patients with values below 300 U/L as normal, those with values between 300 and 1000 U/L as high, and those with values above 1000 U/L as very high [9,11,12]. The patients were categorized into groups based on GGT levels at the time of diagnosis and before and after KPE.

Histology at the time of the KPE was analyzed from liver biopsy, using the Ludwig–Batts classification of fibrosis severity [13]. Post-KPE evolution was established on clinical and laboratory data.

For the statistical analysis, we used Statistica version 13 (TIBCO Software Inc., Palo Alto, CA, USA, 2018). Descriptive statistics were used to summarize continuous variables as means and standard deviations, and categorical variables as proportions and percentages. Group comparisons between patients with low and high GGT levels, as well as between those with favorable and unfavorable outcomes, were performed using Student’s *t*-test for continuous variables and Pearson chi-square test for categorical variables. Receiver operating characteristic (ROC) curve analysis was conducted to assess the predictive value of GGT levels at diagnosis, before, and after KPE for unfavorable outcomes, with area under the curve (AUC) and *p*-values reported. We used the EasyROC web tool (Turkey, version 1.3.1.) available online [14], considering a test clinically useful if its AUC was over 0.7. To compare native liver survival in patients with KPE between those with low and high GGT levels, we used the Kaplan–Meier method and the log-rank test to determine statistical significance. Subgroup analyses were also performed by stratifying patients according to GGT thresholds of 200 U/L and 300 U/L, and clinical and laboratory parameters, as well as outcomes, were compared between these subgroups. Throughout the analysis, *p*-values were reported for all comparisons, with a significance threshold set at 0.05.

This study was conducted in accordance with the principles of the Declaration of Helsinki and with informed consent from the patients’ parents.

## 3. Results

Our study includes 47 children aged between 3 days and 7 months (mean age of 69.11 ± 51.08 days). There were 21 males (44.68%). The average weight at presentation was 3300 g (range: 2500–4000 g). Only two patients (4.25%) had other associated congenital anomalies, including tetralogy of Fallot in one case, atrial and ventricular septal defects in another, and polysplenia and renal dysplasia in both cases. The final evolution of the patients included in the study was unfavorable in 33 patients (70.21%), 19 patients (40.43%) underwent LT, and 14 patients (29.79%) had a fatal outcome.

KPE was carried out in 29 patients (61.70%), of whom 15 were children (51.72%) who had an unfavorable outcome (eight patients received LT, and seven patients died). One infant died immediately postoperatively because of a severe abdominal infection, and six patients progressed to liver cirrhosis and had a fatal outcome without the possibility of performing LT. The mean age at KPE surgery was 7.96 ± 1.94 weeks (range: 2–11 weeks). In the absence of KPE (18 patients; 38.29%), the outcome was unfavorable: 11 patients received LT, while seven died; the majority were social cases without the possibility of receiving LT. As expected, there was a better outcome in patients with KPE (Pearson chi-square, *p* = 0.0010).

The histology of the liver was assessed through a liver biopsy at KPE and staged based on the Ludwig–Batts classification: stage 1, mild fibrosis (portal fibrosis); stage 2, moderate fibrosis (periportal fibrosis or rare portal-to-portal septa); stage 3, severe fibrosis (septa with architectural distortion but not cirrhosis); and stage 4, cirrhosis [13]. Data were available for 25 of the patients with KPE performed: one patient (4%) with mild fibrosis, one patient (4%) with moderate fibrosis, 18 patients (72%) with severe fibrosis and five patients (20%) with cirrhosis. Another five patients without KPE performed had histological data available: two patients with severe fibrosis and three with cirrhosis.

Of the patients with KPE, eight had a favorable evolution after 3 months, and eight others after 6 months from the moment of KPE. Of the 16 with an initial favorable evolution, four ultimately required LT, and two had a fatal outcome. In total, 19 patients received LT at a mean age of 576.05 ± 573.55 days (range: 101–1982 days).

We analyzed laboratory parameters at the time of BA diagnosis, based on patients’ outcomes (Table 1), and found no statistically significant differences in any parameters between those who survived with a native liver and those with LT or a fatal outcome.

The age at diagnosis was lower in patients with a favorable outcome than in those with an unfavorable outcome: 46 ± 36.89 days vs. 78.91 ± 53.53 days (*p* = 0.0421).

**Figure 1:**
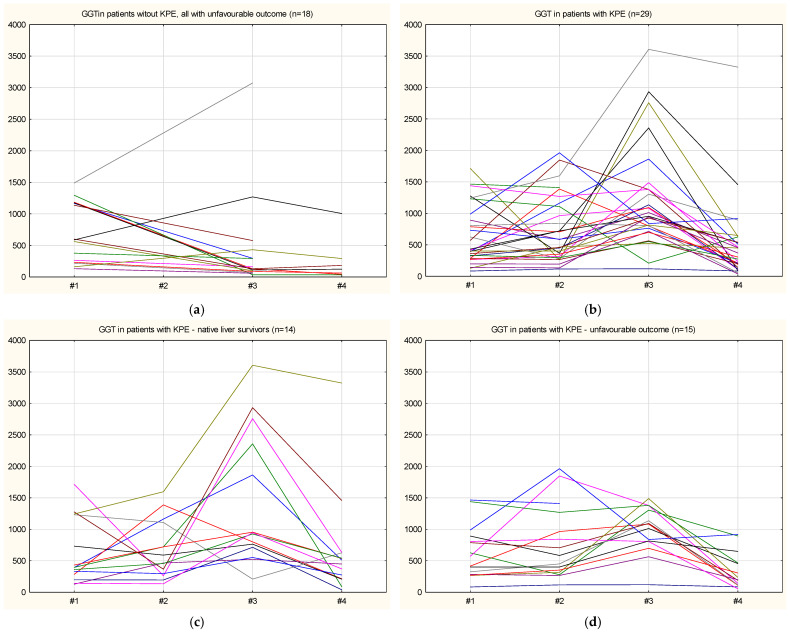
Evolution of GGT serum level in patients without (**a**), and with KPE (**b**), stratified by the outcome, native liver survivors (**c**) or unfavorable (**d**). Note: for patients without KPE: #1, at diagnosis; #3, after three months; #4, after six months. For patients with KPE: #1, at diagnosis; #2, before KPE; #3, after KPE; #4, three months after KPE).

We analyzed the BA patient cohort based on GGT level at diagnosis. In our laboratory, a value of 200 U/L can be considered the upper limit of the normal range. As in the study conducted in China, which set the upper limit of normal at 300 U/L, we also used this value to discriminate between patients based on GGT levels [9]. Figure 1 shows the individual GGT serum level plots over time for patients without KPE (all with unfavorable outcomes) and for patients with KPE, grouped by outcome (native liver survivors and unfavorable outcomes).

The GGT level was below 200 U/L in 6 patients (12.76%) at the time of diagnosis. Comparing patient outcomes by GGT level, no significant differences were found (*p* = 0.2464). Specifically, 3/6 infants with normal GGT levels had a poor prognosis (50%), compared with 30/41 infants with higher GGT levels (73.17%). Of those with normal GGT levels, only 4 infants underwent KPE; one was presented late (at 3 months of age), and in one case, a delay in the final diagnosis led to a late moment for an eventual KPE.

Analysis of age at diagnosis showed no difference by GGT level (51.83 ± 39.64 days in the normal GGT group vs. 71.63 ± 52.47 days in the high GGT group; *p* = 0.3810).

The severity of fibrosis was similar between normal and higher GGT levels (*p* = 0.0768): none vs. one with mild fibrosis, one vs. none with moderate fibrosis, two vs. 18 with severe fibrosis, and one vs. 7 with cirrhosis.

Of the 29 patients with KPE, 4 had normal GGT levels. The age at surgery was not significantly different based on the GGT serum level (7.25 ± 1.26 days vs. 8.08 ± 2.02 days, *p* = 0.4360). The initial favorable evolution was observed in 3 patients with normal GGT levels and in 13 patients with higher GGT levels. All three with a normal GGT level survived with a native liver. One patient with normal GGT presented with failed KPE and ultimately needed LT. From the group of higher GGT level and initial favorable evolution post-KPE, seven survived with native liver (53.85%), 4 needed LT and two died. Overall, for the group in which KPE was performed, a favorable outcome was seen in 3 of 4 infants with normal GGT (75%) compared to 11 of 25 infants with higher GGT (44%, *p* = 0.2493).

For patients who needed LT, the analysis based on GGT level revealed that those with normal GGT had a lower age at LT than those with high GGT, although the difference was not statistically significant (240 ± 29.70 days vs. 615.59 ± 595.19 days, *p* = 0.3964).

Considering the 300 U/L cut-off as the boundary between normal and high GGT levels, we conducted the same analysis. 14 infants (29.78%) had GGT levels under 300 U/L. The age at diagnosis was higher in the group with normal GGT levels, but the effect of GGT level was not statistically significant (77.12 ± 60.21 days vs. 65.70 ± 47.31 days, *p* = 0.4884).

Based on histological examination, the severity of fibrosis was similar between the two groups (*p* = 0.412): none vs. one with mild fibrosis; one vs. none with moderate fibrosis; six vs. 14 with severe fibrosis; and two vs. six with cirrhosis.

Regarding the outcome, 10 of those had an unfavorable outcome (71.43%) compared with 23 of 33 (69.70%) with higher GGT levels (*p* = 0.9055). Among patients with KPE, 8 of 29 had a GGT level below 300 U/L. The age at KPE was similar in both groups, 7.63 ± 1.41 vs. 8.09 ± 2.12 weeks, *p* = 0.5683. The survival rate with native liver was identical in both groups: 50% in those with normal GGT levels, compared with 47.62% in those with higher GGT levels (*p* = 0.9087). Initial evolution post-KPE was favorable in 16 infants, including five from the normal GGT group (5/8, 62.5%) and 11 from the high GGT group (11/21, 52.38%), with no significant difference observed (*p* = 0.6243). Of those five with favorable evolution, only one ultimately required LT, while four survived with a native liver.

In those patients without KPE, six had GGT levels under 300 U/L (four of whom had a fatal outcome and two underwent liver transplantation, LT), and 12 had higher GGT levels (three with a deadly outcome and nine underwent LT), *p* = 0.0874.

Regarding patients who needed LT, five had a GGT level under 300 U/L, and the age at LT was 270.40 ± 147.45 days, which was lower than in those with higher GGT levels but not significant (685.21 ± 632.48 days, *p* = 0.1715). Additionally, when comparing patients with KPE (275.33 ± 208.29 days vs. 1196.40 ± 714.96 days, *p* = 0.0787) and those without KPE (263.00 ± 2.83 days vs. 401.22 ± 374.72 days, *p* = 0.6287), the age at LT was lower in those with normal GGT.

There was no difference in disease severity or outcome across GGT groups, even when stratified into high (300–1000 U/L) and very high (>1000 U/L) categories.

**Figure 2:**
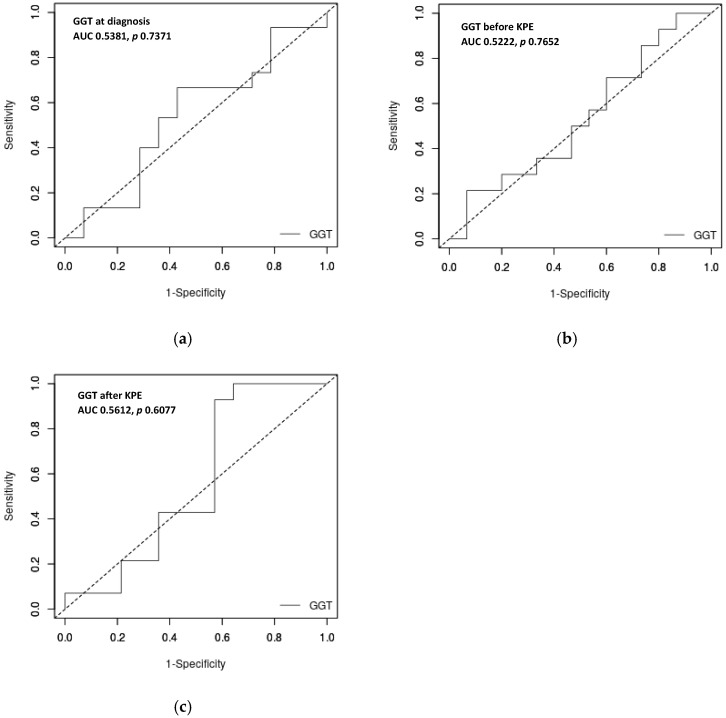
ROC curve for GGT level at diagnosis (**a**), before KPE (**b**), and after KPE (**c**) to predict the unfavorable outcome. None of the ROC curves showed significant predictive value for the outcome.

Analyzing the possible role of a GGT cut-off in predicting outcome, the ROC curve showed that GGT levels at diagnosis, as well as before and after KPE, are not strong predictors of outcome in our cohort of BA patients. The AUCs were 0.5381 (*p*-value = 0.7371) for the GGT level at diagnosis and 0.5222 (*p*-value = 0.7652) for the GGT level before KPE. Upon analyzing the GGT level at the first control after KPE, we reach the same conclusion: AUC is 0.5612, *p* = 0.6077 (Figure 2). All AUCs are under 0.7, and therefore, GGT level is not useful to predict the outcomes.

**Figure 3:**
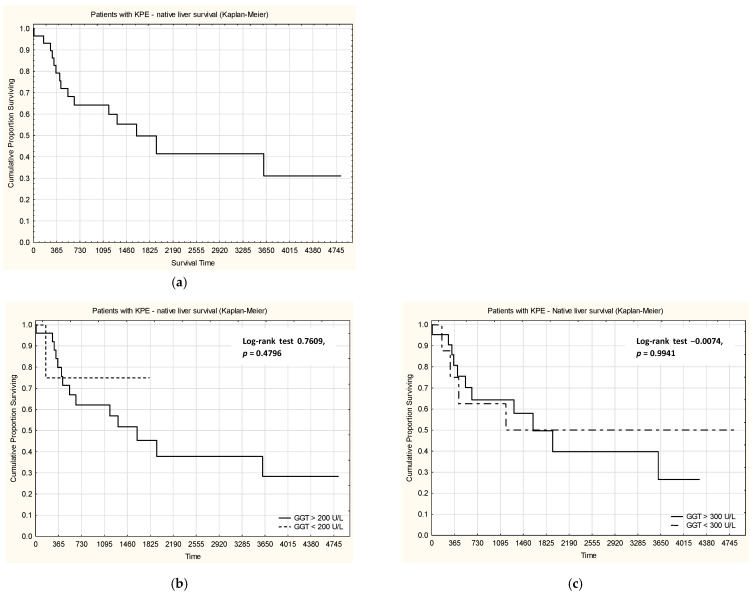
Native liver survival in patients with KPE (**a**), stratified by the serum level of GGT, using 200 U/L (**b**) and 300 U/L as a cut-off (**c**). Note: no statistically significant differences were found.

When analyzing survival with native liver in patients with KPE (Figure 3), no statistically significant differences were observed between patients with GGT levels considered normal or high (using both cut-off values, 200 U/L and 300 U/L).

Multivariate logistic regression analysis included GGT, alkaline phosphatase, TB, DB, ALT, AST, INR, ALB, and age at diagnosis and age at KPE as independent variables. We did not identify GGT serum level or any other parameter as a significant factor for survival with native liver in our cohort (Table 2).

## 4. Discussion

GGT is a biomarker of liver disease and is involved in the metabolism of inflammatory mediators, carcinogenic, and toxic substances. The enzyme is involved in glutathione (GSH) metabolism and has a significant antioxidant effect on cells, protecting them against reactive oxygen species and free radicals. It mediates the transfer of the γ-glutamyl fragment of GSH to other amino acids and facilitates the passage of substances across cell membranes [1]. Thus, GGT plays a crucial role in GSH recycling and promotes the binding of various exogenous substances (such as drugs and toxins) or endogenous substances (including leukotrienes and prostaglandins), thereby stimulating their cellular metabolism and excretion [1].

GGT is located on the outer surface of cells with intense detoxification activity, such as hepatocytes, cholangiocytes, proximal tubular cells, and pancreatic ductal cells. Smaller quantities are present in cardiomyocytes, platelets, neurons, glial cells, bronchial and alveolar epithelial cells, and reproductive cells [12]. In the liver, GGT is mainly located in the capillary area of hepatocytes, on their plasma membrane, and is particularly concentrated on the cholangiocytes’ membrane and in the cells surrounding the bile ducts [2]. The bile canaliculi are formed by the tight junctions between the apical canalicular membranes of adjacent hepatocytes, which surround, except for the sinusoidal area. The bile secreted by the hepatocytes circulates through the canaliculi towards the center of the hepatic lamina, is collected in the intrahepatic bile ducts that converge to the extrahepatic ducts and finally to the main duct [15,16]. Any abnormality in bile formation or flow impairment is abnormal and may be a sign of a serious hepatobiliary disorder. An important cause of cholestasis is cholangiopathy, which may be immune-mediated or due to genetic, infectious, toxic, vascular, or obstructive causes. Immune-mediated cholangiopathies comprise primary sclerosing cholangitis (PSC), autoimmune cholangitis, and IgG4-associated cholangitis in adults, as well as BA and neonatal sclerosing cholangitis (NSC) in children.

The GGT level can vary by age and gender. It is highest in infants younger than 1 month of age (156.7 ± 98.2 U/L), then decreases with age, reaching a lower value that is maintained into adulthood, around 7 months of age [17,18]. In infants younger than 6 months, GGT levels range from 12 to 122 U/L. After this age, the reference range is 1–39 U/L [17,18]. In neonates, GGT levels vary with gestational age, being higher in preterm than in full-term infants (156.7 ± 98.2 U/L) [17]. Male infants show slightly higher GGT values than female infants (145.98 ± 93.68 U/L vs. 132.18 ± 78.97 U/L), probably due to the hormonal influences on the bilirubin conjugation process [19].

The objective of our retrospective study was to investigate GGT as a prognostic marker in immune-mediated cholangiopathies in infancy, especially those with cholangiocyte senescence as the main etiopathogenetic mechanism (BA, NSC, and PSC). These three entities have many similarities, from histopathology or clinical manifestations, but what connects them in particular is a premature or accelerated senescence process of the cholangiocytes leading to the superactivation of the immune system, followed by chronic inflammation, fibrosis and lumen stenosis of the biliary ducts (extrahepatic in BA, intra and extrahepatic in NSC or PSC) lumen stenosis, and finally progressing to cirrhosis [20]. We analyzed data from 47 children with BA diagnosed in our unit to assess the role of GGT serum levels as prognostic markers of BA severity and outcome, with or without KPE and subsequently LT. In our cohort, the patients with KPE had better outcomes, as expected. The mean age at diagnosis was lower in those with a favorable outcome, but some patients with late presentation found KPE infeasible. Analyzing different laboratory parameters, the outcomes of BA patients were not associated with any of the frequently used parameters, except for albumin level, with near statistical significance. Still, as the GGT level was reported as a possible marker for severity and outcome, we analyzed our patients’ data based on this parameter.

BA is a progressive inflammatory cholangiopathy secondary to immune dysregulation associated with a viral infection, with consequences including extrahepatic bile duct fibrosis, impairment of bile flow, and ultimately liver cirrhosis [20,21]. Early diagnosis of infants with BA is crucial, given the need for early surgical intervention (KPE) before 60 days of age [20,21,22,23]. Moreover, the latest studies report that early diagnosis of BA before 30 to 45 days of age, with prompt surgical treatment, increases the likelihood of restoring bile flow and improving the disease’s prognosis. Only a few infants (<20%) in whom KPE was performed late (over 90 days) achieve bile drainage, with the majority progressing to cirrhosis and requiring LT [21,24].

The correlation between BA and elevated GGT levels is well known. The accuracy of GGT in diagnosing BA at different cut-off levels varies [25]. The localization of GGT in bile duct epithelial cells explains the marked increase in this parameter in BA, making it a crucial factor in diagnosis, along with specific ultrasound changes [26]. This process is secondary to intrahepatic bile ductular proliferation or periportal hepatocytes metaplasia, the main features of BA, which evolve concomitantly with the progression of extrahepatic bile duct destruction [27].

GGT is an important marker in differentiating BA from other causes of neonatal cholestasis [28,29]. Usually, an elevated GGT could indicate particularly extrahepatic obstructive lesions (BA) and those involving intrahepatic bile ducts (alpha 1 antitrypsin deficiency, cystic fibrosis, Alagille Syndrome), while a normal or low GGT could indicate BASD or PFIC type 1 or type 2 [30]. El-Guindi et al. used a predictive score to distinguish BA from other causes of cholestasis, including GGT levels. The results showed a significantly higher level in BA patients than in other cholestatic disorders. A GGT level > 286 U/L had 76.7% sensitivity and 80% specificity for discriminating between BA and non-BA cholestatic disorders [25]. Another study from China supports the use of GGT levels as a diagnostic test for BA in infants younger than 120 days [26]. Other authors analyzed the GGT-to-transaminase ratio and concluded that a ratio greater than 2 could be a good indicator of BA diagnosis and could shorten the time to KPE [29].

In patients with low GGT, this is explained by severe fibrotic destruction of the extrahepatic ducts, which reduces GGT production by secretory bile epithelial cells (BECs) and its release into the serum. Histopathological studies support this hypothesis, revealing a near-complete ductular obliteration in 70% of low GGT cases [31]. This “burnt-out” phenotype suggests rapid disease progression, leaving fewer viable BECs to secrete GGT [5].

A high GGT level before KPE is usually associated with a better prognosis after surgery, a higher rate of jaundice clearance, and a lower mortality. GGT and its metabolite, glutathione, generate free radicals that have pro-oxidative effects on the cholangiocyte membrane, triggering inflammation and fibrosis. Therefore, the increase in GGT in patients with BA without jaundice after KPE can be explained not only by postoperative biliary obstruction, but also by ongoing progressive liver fibrosis. Long-term follow-up of patients with BA has shown that serum GGT levels may indicate progression to fibrosis and portal hypertension even if the KPE was successful and bile flow was restored. Dong et al. reported that serum GGT levels are well correlated with persistent local inflammation. Acute and chronic inflammatory processes in the extrahepatic bile ducts have been demonstrated by increased IL-33 expression in serum and liver tissue of patients after KPE surgery. IL-33, along with Connective Tissue Growth Factor (CTGF, secreted by fibroblasts after activation by TGF-β1), is an important proinflammatory cytokine that contributes to increased oxidative stress and liver fibrosis. Exposure of cholangiocytes to these cytokines will increase oxidative stress and liver fibrosis, even in the absence of jaundice [32]. Another study of 169 Korean infants who underwent KPE found that serum GGT concentrations greater than 550 U/L at month 5 after KPE were significantly associated with worse outcomes and lower survival with a native liver [7].

But in some infants with BA, serum GGT levels remain within the normal range (often <150 U/L), despite severe cholestasis. This could also be due to the delay in establishing the diagnosis of BA, considering that the genetic causes of neonatal cholestasis are well known. These include low GGT as a cause of cholestasis, especially BA, and PFIC-2 (ABCB11 gene variants). In Melbourne, out of 113 infants diagnosed with BA between 1991 and 2017, 30% underwent unnecessary genetic testing, delaying KPE by 10–14 days, because they had low GGT [8]. Biliary Atresia Splenic Malformation (BASM) syndrome, a form of embryonal BA associated with splenic malformation (polysplenia), cardiac and vascular anomalies, or heterotaxia, often evolves with low or normal GGT [33].

Around 12–14% of infants with BA had normal GGT levels at presentation. This association is characterized by a severe progression of the disease, even if the diagnosis is made early [8]. In patients with low GGT, glutathione reserves are depleted due to intense oxidative stress following the accumulation of toxic bile acids. Patients with low GGT appear to have a distinct genetic phenotype, characterized by a high expression of polymorphisms in glutathione metabolism genes (*GSTP1* c.313A>G) that impair glutathione conjugation and exacerbate oxidative injury [34,35].

A large study from Shanghai, China, reinforces the idea that a low GGT level at diagnosis can predict a more severe course of the disease. Of the 1998 children admitted with BA over six years, 496 had low GGT levels (<300 IU/L, according to the hospital’s reference range) [9]. In the preoperative period, most of them had an unfavorable evolution despite the precocious diagnosis [9]. Low GGT patients presented with more advanced histopathological injury: 80% with METAVIR (Meta-analysis of Histological Data in Viral Hepatitis) stage F4 fibrosis at diagnosis, compared to 40% in high GGT BA [9]. These patients also exhibited worse coagulation profiles, reflecting impairment of hepatic synthesis function [9]. Post-KPE intervention, infants with low GGT BA had a significantly lower jaundice clearance rate at 6 months and reduced 2-year native liver survival [9]. Therefore, some authors report that low GGT levels may serve as a prognostic marker for disease progression [8]. Similar results were observed in Anhui Province, China, where 74 infants with BA were analyzed. A low level of GGT before the KPE was associated with a poor outcome, suggesting it may be a marker of an unfavorable prognosis [36]. Moreover, children with low or normal GGT levels at diagnosis received liver transplantation at a younger age and had a significantly lower native liver survival rate than those with high GGT [9].

In our cohort, we attempted to replicate the Chinese study’s results using a 300 U/L cut-off for patients with normal or low GGT levels [9]. We included all patients with BA, not only those with KPE, as in the Chinese study. They also excluded patients who were not followed up long-term during the early period of their research, which may introduce bias. Anyway, the large number of patients included in the Chinese study made their results reliable [9]. In our cohort, one-third of our patients had serum GGT levels below 300 U/L, similar to the Chinese study (24.8%). In contrast to their results, we did not find significant differences based on the GGT levels. The age at diagnosis was slightly higher, but the difference was not statistically significant. There was no significant difference in fibrosis severity or age at KPE. Even though the age at LT was lower in those with lower GGT levels in both KPE patients and non-KPE patients, the difference was not statistically significant. Overall, in our cohort, the outcome (death, LT, or survival with native liver) was not influenced by GGT level at diagnosis.

In Australian children with BA, a normal GGT at presentation was associated with an unfavorable outcome. Among children diagnosed with BA, 12.3% had normal GGT values at diagnosis (<200 U/L, compared with the Australian Center laboratory reference values). They required LT sooner than those with high GGT at diagnosis and had poorer transplant-free survival. The authors also remind us not to be too prescriptive about GGT levels when investigating an infant with jaundice to rule out BA, as we discuss later [8].

When we used 200 U/L as the normal limit for GGT, we observed a similar proportion of patients as in the Australian study. The age at diagnosis and at KPE were similar across groups based on the GGT level. The severity of fibrosis was not correlated with GGT levels. Among patients with KPE, those with lower GGT had better outcomes, but the difference was not statistically significant. As in the Australian study, the age at LT was lower among our patients who had this opportunity, particularly those with lower GGT levels. In this group, the LT was performed at a mean age of 8 months. Although the differences were not statistically significant, this may be due to disease severity, but the low number of patients may be the reason for the lack of significance. We consider that this limit of 200 U/L is closer to the upper limit of normal values in many laboratories and may also be used for this analysis in other studies.

We did not find any published cases or cohort studies on low or normal GGT at diagnosis of BA in North America; most publications from the USA or Canada emphasize the utility of high GGT levels as part of the diagnosis of BA and their importance in predicting prognosis after KPE [3,37,38,39]. The medical reports of 219 children with BA living with their native livers 5 years or more after KPE were analyzed by the North American Multicentre Consortium, with the results being of great interest. More than 85% of patients have biochemical evidence of chronic liver disease even without clinical abnormalities and without cholestasis, with an approximately normal median level of GGT [40].

We sought to identify a possible cut-off for GGT levels that could serve as a prognostic marker for disease outcome. We analyzed GGT levels at diagnosis and in those with KPE at the time of surgery and after surgery. In all these analyses, the AUCs were not high enough to be significant and useful for predicting outcomes based on the GGT levels. Based on data from our real-life cohort, including all patients without prior selection, we cannot demonstrate differences in BA severity or outcome by GGT serum levels.

Anyway, in our unit, if there are clinical, laboratory, and imaging data supporting the diagnosis of BA, we did not change our approach based on GGT levels. In our cohort, there was only one case, among the earliest, in which a low GGT level delayed the diagnostic approach, necessitating consideration of alternative genetic causes of cholestasis. Due to this delay, the proper time for KPE was overcome. Since this case, which proved to be BA, we have been referring patients to surgeons as soon as possible. Probably because of this approach, we did not observe differences in time to diagnosis or time to KPE by GGT level.

In real life, some patients with BA present later to the hepatology unit at an age that limits the KPE surgery and with severe fibrosis. Mainly, these are patients coming from socially deprived regions without proper medical care. In this BA patient category, due to severe fibrosis, some patients may present with lower GGT levels, potentially biasing statistical analysis.

Based on our experience, we recommend pursuing a timely diagnostic approach in suspected BA patients and not delaying testing for BASD or PFIC in those with lower GGT levels (though the test can be performed promptly). The availability of genetic tests and the decrease in time to results supported us in difficult cases. Even though in some countries these genetic tests are not yet available, the European initiative to create networks among hepatology centers helps all specialists and patients access proper diagnosis and care [41]. Regarding the post-KPE evaluation, we recommend that, in patients with lower GGT levels, follow-up focus on early referral to LT due to a probable higher disease severity (as shown by other studies, not necessarily ours, aside from the lower age at LT). Also, in patients without KPE, LT evaluation should be earlier in those with lower GGT levels. We based this affirmation on our data, which showed a higher fatality rate without LT in 4 of 6 with lower GGT levels compared with 3 of 12 with higher GGT levels (not statistically significant).

The limitations of our study stem from its single-center design and limited patient numbers. The second issue is that some of the patients presented late, and with already higher stages of fibrosis that could be linked with low GGT levels. For some patients referred late from other services, we did not have laboratory data from the time of their first suspicion of cholestasis. Not all of our patients had fibrosis evaluated at the time of KPE, and for those without KPE, we did not use the same histologic method to assess fibrosis. We considered only histological data from liver biopsy, as elastography in cholestasis may be biased. This decision limited the significance of the correlation between GGT levels and fibrosis severity. To better assess the possible role of GGT levels in predicting outcomes, studies including more patients or multicenter studies using the same approach must be performed. Otherwise, conclusions may be based on limited studies or case reports that may overlook considerations of late presentation and severe fibrosis resulting from delayed evaluation.

## 5. Conclusions

While elevated GGT is a hallmark of BA, in our cohort, 12% of cases present with normal or low GGT levels, a paradoxical finding associated by some experts with severe disease and unfavorable evolution. Our study did not show that lower GGT levels are clearly linked to late diagnosis, late age at KPE, or different outcomes. Only the time to LT was earlier in patients with lower GGT, but this difference was not statistically significant. Few studies have analyzed the role of GGT levels, and, in contrast with other data, our study raises concerns about previous reports. We did not find any GGT level measured at diagnosis, at the KPE moment, or after surgery that could be used to predict the outcome. We recommend proceeding with a timely diagnostic approach without delay based on GGT levels in patients with high suspicion of BA, and referring patients to KPE as early as possible. After KPE, it would be better to evaluate patients with lower GGT levels early for LT referral to improve outcomes.

## Figures and Tables

**Table 1:** Laboratory parameters at the diagnosis, based on the outcome.

Variable	All Patients *n* = 47	Favorable Outcome *n* = 14	Unfavorable Outcome *n* = 33	*p*-Value
GGT (U/L)	639.89 ± 455.69	633.71 ± 514.60	642.51 ± 436.93	0.9525
Alkaline Phosphatase (U/L)	658.38 ± 378.77	623.64 ± 351.93	673.12 ± 393.92	0.6868
TB (mg/dL)	10.14 ± 3.29	10.08 ± 2.94	10.16 ± 3.47	0.9373
DB (mg/dL)	7.37 ± 2.68	6.42 ± 1.90	7.78 ± 2.89	0.1143
ALT (U/L)	156.81 ± 126.89	121.64 ± 93.12	171.73 ± 137.30	0.2196
AST (U/L)	250.26 ± 173.20	210.86 ± 159.18	266.97 ± 178.50	0.3150
INR	1.54 ± 1.81	1.00 ± 0.13	1.75 ± 2.11	0.2138
Albumin (g/dL)	3.64 ± 0.39	3.79 ± 0.31	3.58 ± 0.40	0.0842

Note: No statistically significant differences were found for favorable and unfavorable outcome groups. GGT, gamma-glutamyl transpeptidase; TB, total bilirubin; DB, direct bilirubin; ALT, alanine aminotransferase; AST, aspartate aminotransferase; INR, international normalized ratio.

**Table 2:** Multivariate logistic regression analysis of the correlation between parameters and survival with native liver in BA patients.

Variable	Estimate	SE	Wald	Lower CI 95%	Upper CI 95%	*p*-Value
GGT	−0.000499	0.001582	0.099560	−0.003600	0.002601	0.7523
Alkaline Phosphatase	−0.001136	0.004515	0.063357	−0.009987	0.007714	0.8012
TB	1.339435	0.761421	3.094517	−0.152923	2.831794	0.0785
DB	−2.839860	1.479841	3.682675	−5.740296	0.060576	0.0549
ALT	−0.026695	0.020257	1.736697	−0.066398	0.013007	0.1875
AST	0.026418	0.018860	1.962070	−0.010547	0.063384	0.1612
INR	−2.029420	7.584581	0.071594	−16.894928	12.836086	0.7890
Albumin	4.903228	2.944397	2.773137	−0.867684	10.674140	0.0958
Age at diagnosis	−0.040426	0.034292	1.389794	−0.107638	0.026784	0.2384
Age at KPE	−0.039902	0.077656	0.264032	−0.192105	0.112300	0.6073

Note: No significant factor for survival with native liver was identified. GGT, gamma-glutamyl transpeptidase; TB, total bilirubin; DB, direct bilirubin; ALT, alanine aminotransferase; AST, aspartate aminotransferase; INR, international normalized ratio; KPE, Kasai portoenterostomy; SE, standard error.

## Data Availability

The raw data supporting the conclusions of this article will be made available by the authors upon request.

## Abbreviations

The following abbreviations are used in this manuscript:

ALT	Alanine Aminotransferase
AST	Aspartate Aminotransferase
AUC	Area Under the Curve
BA	Biliary Atresia
BASD	Bile Acid Synthesis Disorders
BASM	Biliary Atresia Splenic Malformation Syndrome
BEC	Bile Epithelial Cells
CTGF	Connective Tissue Growth Factor
DB	Direct Bilirubin
GGT	Gamma-Glutamyl Transpeptidase
GSH	Glutathione
IL-33	Interleukin-33
INR	International Normalized Ratio
KPE	Kasai hepatportoenterostomy
LT	Liver Transplantation
METAVIR	Meta-analysis of Histological Data in Viral Hepatitis
NSC	Neonatal Sclerosing Cholangitis
PFIC	Progressive
PSC	Primary Sclerosing Cholangitis
ROC	Receiver operating characteristic
TB	Total bilirubin
TGF-β1	Transforming Growth Factor-beta 1
